# Possible Alternatives: Identifying and Quantifying Adulteration in Buffalo, Goat, and Camel Milk Using Mid-Infrared Spectroscopy Combined with Modern Statistical Machine Learning Methods

**DOI:** 10.3390/foods12203856

**Published:** 2023-10-21

**Authors:** Chu Chu, Haitong Wang, Xuelu Luo, Peipei Wen, Liangkang Nan, Chao Du, Yikai Fan, Dengying Gao, Dongwei Wang, Zhuo Yang, Guochang Yang, Li Liu, Yongqing Li, Bo Hu, Zunongjiang Abula, Shujun Zhang

**Affiliations:** 1 Frontiers Science Center for Animal Breeding and Sustainable Production, Ministry of Education, Huazhong Agricultural University, Wuhan 430070, China; chu1999@webmail.hzau.edu.cn (C.C.); htw0411@webmail.hazu.edu.cn (H.W.); lxl775282323@163.com (X.L.); wenpeipei@webmail.hzau.edu.cn (P.W.); 15827557518@163.com (L.N.); dc1992hml@163.com (C.D.); fanyikai123@webmail.hzau.edu.cn (Y.F.); wangdwei@webmail.hzau.edu.cn (D.W.); yang_zhuo@webmail.hzau.edu.cn (Z.Y.); yangguochang726@webmail.hzau.edu (G.Y.); liuli17509991115@webmail.hzau.edu.cn (L.L.); liyongqing@webmail.hzau.edu.cn (Y.L.); 2 Key Laboratory of Animal Genetics, Breeding and Reproduction, Ministry of Education, College of Animal Science and Technology, Huazhong Agricultural University, Wuhan 430070, China; gdyy0107@163.com; 3 Quality Standards Institue of Animal Husbandry, Xinjiang Academy of Animal Science, Urumqi 830012, China; hubo123@sina.cn (B.H.); 17509991115@163.com (Z.A.)

**Keywords:** mid-infrared spectroscopy, machine learning, milk adulteration, milk

## Abstract

Adulteration of higher priced milks with cheaper ones to obtain extra profit can adversely affect consumer health and the market. In this study, pure buffalo milk (BM), goat milk (GM), camel milk (CM), and their mixtures with 5–50% (vol/vol) cow milk or water were used. Mid-infrared spectroscopy (MIRS) combined with modern statistical machine learning was used for the discrimination and quantification of cow milk or water adulteration in BM, GM, and CM. Compared to partial least squares (PLS), modern statistical machine learning—especially support vector machines (SVM), projection pursuit regression (PPR), and Bayesian regularized neural networks (BRNN)—exhibited superior performance for the detection of adulteration. The best prediction models for the different predictive traits are as follows: The binary classification models developed by SVM resulted in differentiation of CM-cow milk, and GM/CM-water mixtures. PLS resulted in differentiation of BM/GM-cow milk and BM-water mixtures. All of the above models have 100% classification accuracy. SVM was used to develop multi-classification models for identifying the high and low proportions of cow milk in BM, GM, and CM, as well as the high and low proportions of water adulteration in BM and GM, with correct classification rates of 94%, 100%, 100%, 99%, and 100%, respectively. In addition, a PLS-based model was developed for identifying the high and low proportions of water adulteration in CM, with correct classification rates of 100%. A regression model for quantifying cow milk in BM was developed using PCA + BRNN, with RMSEV = 5.42%, and R_V_^2^ = 0.88. A regression model for quantifying water adulteration in BM was developed using PCA + PPR, with RMSEV = 1.70%, and R_V_^2^ = 0.99. Modern statistical machine learning improved the accuracy of MIRS in predicting BM, GM, and CM adulteration more effectively than PLS.

## 1. Introduction

With the growing demand for food and the increasing globalization of the supply chain, food quality and safety have become a growing concern for consumers, food producers, and governments. Food adulteration is defined as the process of intentionally lowering the quality of a food product by adulterating it with low-quality materials or extracting valuable ingredients from it [1]. Milk is one of the food products most susceptible to adulteration [2], where raw milk adulteration is a recurring problem in many countries. The value and price of buffalo, goat, and camel milk are higher than that of cow milk [3,4], which suggests the possibility that some producers may be able to increase their profit margins by adulterating high-value milk using cow milk or water. Apart from the ethical, religious, and cultural implications, raw milk adulteration has adverse effects on the health and property safety of consumers, as the milk components in these adulterated products may induce allergies and other adverse reactions [5]. Due to differences in composition and sensory attributes, goat milk and buffalo milk are the preferred types of milk for some cheeses and yogurts [6]. The addition of cow milk to buffalo or goat milk affects the processing conditions and the final quality of the dairy product, including texture and sensory properties, which can have a negative impact on the dairy market [7]. Therefore, there is a need to develop a rapid and reliable method to detect the quality and authenticity of raw milk in order to protect consumers and the dairy industry.

Currently, some methods have been developed to detect possible fraudulent milk or water adulteration in buffalo milk, goat milk, and camel milk, such as species-specific polymerase chain reactions, an enzyme-linked immunosorbent assay, capillary electrophoresis [6], polyacrylamide gel electrophoresis, and high-performance liquid chromatography [8]. Isoelectric focusing of fibrinolytic γ casein is the official method for detecting the presence of cow milk in goat milk and buffalo milk [9]. All of the above techniques have some limitations, such as being time-consuming, costly, generating large quantities of chemical waste, not being able to detect on a large scale, and the need for specialized personnel, which prevents these tools from being used for large-scale screening of raw milk adulteration in the dairy industry. In recent years, increasing attention has been paid to the application of spectroscopic techniques, namely vibrational spectroscopy, infrared spectroscopy, and Raman spectroscopy in food, animal science, and agriculture [10]. Attenuated total reflectance Fourier-transform infrared spectroscopy, Raman spectroscopy, near-infrared spectroscopy, and laser-induced breakdown spectroscopy have been used for detecting the adulteration in buffalo milk, goat milk, and camel milk [4,11,12,13,14], and have shown good predictive potential. Mid-infrared spectroscopy (MIRS) is a real-time, sensitive, fast, green, high-throughput, clean, and low-cost biochemical fingerprinting technique that does not require sample preparation, and can give results within one minute [15]. The spectroscopic technique is based on the study of the interaction between matter and electromagnetic radiation. In the mid-infrared region (2500 to 25,000 nm), when matter is crossed by electromagnetic radiation, the bonds of the molecules make movements (e.g., vibration and rotation) through molecular bonds, which results in varying degrees of energy absorption. By analyzing the energy supplied and the energy absorbed by the sample, the chemical composition of the sample under test can be determined [16,17]. MIRS, combined with appropriate machine learning algorithms, can extract qualitative and quantitative information from spectra, and thus reach rapid characterization, classification, and quantitative prediction of food products. Therefore, MIRS technology may be an ideal solution for detecting and quantifying adulteration (e.g., adulteration with cow milk or water) in these milks. Several studies have also demonstrated the effectiveness and potential of MIRS in predicting adulteration in buffalo milk [7,18], goat milk [3,7], and camel milk [19,20], but few studies report the application of MIRS in predicting water adulteration in buffalo milk, goat milk, and camel milk.

The reliability and accuracy of MIRS prediction results are highly dependent on model quality, as well as the modeling dataset, spectral quality, and algorithms (including variable selection, spectral preprocessing, and model) used to develop the predictive model [21]. Partial least squares (PLS) is the preferred and most traditional way to correlate MIRS data with milk and animal traits because of its ability to consider covariate and high-dimensional datasets. However, for complex relationships between variables (e.g., nonlinearities and interactions), PLS may not be an ideal treatment [22]. Some MIRS studies on milk have demonstrated that other machine learning algorithms such as random forests (RF), decision trees, and neural networks (NN) are also able to effectively handle milk MIRS data [23]. They are able to construct a model for complex relationships, but to date, these modern statistical machine learning algorithms are not commonly used in MIRS analysis. Furthermore, few studies have explored their potential to utilize MIRS information to predict adulteration in raw animal milk, as well as comparisons with PLS algorithms. Therefore, the aim of this study was to (1) investigate the effectiveness of modern statistical machine learning algorithms combined with MIRS in identifying and quantifying adulteration (adulteration with cow milk or water) of raw animal milk (buffalo milk, goat milk, and camel milk); (2) compare the performance of various modern statistical machine learning algorithms using PLS as a baseline and a control, and determine the optimal algorithm; (3) develop classification models and regression models for detecting the adulteration of cow milk or water in buffalo milk, goat milk, and camel milk, based on optimal MIRS preprocessing and optimal modeling algorithms.

## 2. Materials and Methods

### 2.1. Milk Samples

In total, 157 Holstein cow milk, 198 buffalo milk, 40 goat milk, and 97 camel milk samples were collected from China between January and December 2021 during the morning milking. Buffalo breeds include Mediterranean buffalo, Niliravi buffalo, Mulla buffalo, and crossbred buffalo. Milk samples were collected in batches and immediately delivered to the laboratory of Huazhong Agricultural University for preparation of adulterated samples after each collection (store at 4 °C).

Cow milk was added to buffalo milk, goat milk, and camel milk, and mixed in the following proportions: (1) proportion of cow milk in buffalo milk: 0%, 5%, 10%, 20%, and 50% (vol/vol); (2) proportion of cow milk in goat milk and camel milk: 0%, 20%, and 50% (vol/vol). Similarly, distilled water was added to buffalo milk, goat milk, and camel milk, and mixed in the following proportions: (1) proportion of water in buffalo milk: 0%, 5%, 10%, 20%, 40%, and 50% (vol/vol); (2) proportion of water in goat milk and camel milk: 0%, 20%, and 50% (vol/vol). Not every sample had both pure milk–cow milk mixtures and pure milk–water mixtures due to sample volume limitations. Fresh pure milk and adulterated samples were kept refrigerated at 4 °C and immediately delivered to the DHI center and analyzed using the MilkoScan FT+ (Foss, Hillerød, Denmark) instrument to obtain the MIRS and the chemical composition of the samples.

A total of 224 buffalo milk–cow milk mixtures, 417 buffalo milk–water mixtures, 80 goat milk–cow milk mixtures, 52 goat milk–water mixtures, 165 camel milk–cow milk mixtures, and 181 camel milk–water mixtures were prepared during a period from January to December 2021. The number of samples corresponding to each adulteration ratio is shown in Table 1.

### 2.2. Data Analysis

#### 2.2.1. Spectral Pretreatment

To obey Beer’s law, the spectra were transformed from transmittance into absorbance before modeling. The region from 2968 to 5008 cm^−1^ was considered as noise and removed from the data set. The region from 1773 to 2802 cm^−1^ contained no valuable information and was also removed, together with the saturated water signal (O–H bend) from 1692 to 1604 cm^−1^ [24]. Finally, the remaining 244 wave points were used for modeling (2968 to 2802 cm^−1^, 1773 to 1692 cm^−1^, and 925 to 1604 cm^−1^). Spectral pretreatments are frequently applied to MIRS data to achieve robust prediction models [25]. In this research, the MIRS spectra values were processed using four spectral pre-processing algorithms, i.e., Savitzky–Golay convolution smoothing (SG), first-order derivative (1D), second-order derivative (2D), and standard normal variate (SNV). The R package “prospect” was utilized for the preprocessing steps.

#### 2.2.2. Machine Learning Algorithms

The dataset was randomly divided into a calibration set (80%) and a validation set (20%). The calibration set was used to develop the model, and the validation set was used to validate the performance and generalization ability of the model independently of the calibration set. Three types of models were involved in this study. (1) Binary classification models: pure buffalo milk, goat milk, and camel milk samples were defined as negative 0, and milk–cow milk mixtures or milk–water mixtures were defined as positive 1. The models can be used to identify milk–cow milk mixtures and milk–water mixtures. (2) Multi-classification models: pure buffalo milk, goat milk, and camel milk samples were defined as 0, samples with less than 25% (vol/vol) cow milk or water adulteration were defined as 1, and samples with more than 25% (vol/vol) cow milk or water adulteration were defined as 2. This type of model can be used to classify the samples as no adulteration, low proportion of adulteration, and high proportion of adulteration; (3) Quantitative regression models: these models were established using only buffalo milk data, and can be used to predict the proportion (vol/vol) of cow milk or water added to buffalo milk. The data distribution of each type of model on the calibration and validation sets is shown in Table 2.

Three classification machine learning methods—partial least squares discriminant analysis (PLSDA) and support vector machines (svmLinear and svmRadial)—were used to construct the qualitative model separately, which were then compared with one another. Twelve regression machine learning methods—partial least squares regression (PLSR), Support Vector Machines (svmLinear and svmRadial), Bayesian regularized neural networks (BRNN), spike and slab regression (SSR), projection pursuit regression (PPR), classification and regression tree (CART), ridge regression (RR), least absolute shrinkage and selection operator (LASSO), elastic net regression (EN), RF, and gradient boosting machine (GBM)—were used to construct the quantitative model separately, which were then compared with one another. For all models, the spectral data were scaled and centered before computation so that the mean was equal to 0 and the standard deviation was equal to 1. This procedure was performed using preProc = c (“center”, “scale”) as an argument in the train function of the CARET package. All the machine learning algorithms utilized the CARET package in the R program. All analyses in the present study were performed with R statistical software version 4.2.2 [26]. The inner cross-validation used tenfold cross-validation repeated 5 times, and was used in the analysis subset to tune the optimized hyperparameters and construct the predictive model. Finally, each machine learning method constructed 50 models. For regression models, we selected the model with the lowest root mean square error (RMSE) of cross-validation; For binary classification models, we selected the model with the highest area under the curve (AUC) of cross-validation; For multi-classification models, we selected the model with the highest accuracy of cross-validation.

The maximum number of PLS latent variables was set to 20. The number of the hidden layer for the BRNN varied from 1 to 4. The number of the mtry for the RF was 3, 10, 20, 50, 100, 300, 700, 1000, and 2000. The computation of SVM was based on the support vector machine with a kernel or radial basis function kernel, and was implemented using the method = “svmLinear” or “svmRadial” as arguments in the train function of the CARET package. For “svmLinear”, the tested C values were 0.01, 0.05, 0.1, 0.25, 0.5, 0.75, 1, 1.25, 1.5, 1.75, 2, and 5. For the “svmRadial” kernel, the tested values were 0.01, 0.05, 0.1, 0.25, 0.5, 0.75, 1, 1.25, 1.5, 1.75, 2, and 5 for C; the tested values were 0.01, 0.02, 0.03, 0.04, 0.05, 0.06, 0.07, 0.08, 0.09, 0.1, 0.25, 0.5, 0.75, 0.90 for the sigma.

#### 2.2.3. Performance Evaluation Methods and Metrics

We have used both threshold-independent and threshold-dependent performance evaluation metrics for the evaluation of binary classification models. These included accuracy (Equation (1)), sensitivity (Equation (2)), specificity (Equation (3)), positive predictive value (Equation (4)), and negative predictive value (Equation (5)). Receiver operating characteristic (ROC) curves represent the relationship between a model’s true positive rate and the false positive rate, for different classification thresholds. The AUC measures the area under the ROC curve.

Accuracy = (TP + TN)/(TP + TN + FP + FN)(1)

Sensitivity = TP/(TP + FN)(2)

Specificity = TN/(TN + FP)(3)

Positive predictive value (PPV) = TP/(TP + FP)(4)

Negative predictive value (NPV) = TN/(TN + FN)(5)

True positive (TP) means that an adulterated sample is correctly classified as adulterated, true negative (TN) means that an unadulterated sample is correctly classified as unadulterated, false negative (FN) means that an adulterated sample is incorrectly classified as pure sample, and false positive (FP) means that a pure sample is incorrectly classified as adulterated.

For the multi-classification models, the classification methods’ performance was assessed by the accuracy and kappa value.

The performance of each regression method was evaluated by examining RMSE, mean absolute error (MAE), and the coefficient of determination (R^2^). Furthermore, the ratio of performance to deviation (RPD) was used to assess the model consistency and performance. The RPD is calculated as the ratio of the standard deviation of the reference data to the standard error of prediction. El Jabri et al. summarized the R^2^ and RPD of the prediction equations: (1) Model robustness can be classified into four classes based on the R^2^ value: poor (R^2^ < 0.66), approximate (0.66 < R^2^ < 0.81), good (0.82 < R^2^ < 0.90), and excellent (R^2^ ≥ 0.91). (2) The higher the RPD, the better the model. Models with RPD > 2 can achieve high accuracy predictions. Based on the RPD values, the models can be categorized into six classes of robustness: very poor (RPD < 1), poor (1 < RPD < 1.4), fair (1.4 < RPD < 1.8), good (1.8 < RPD < 2), very good (2 < RPD < 2.5), and excellent (RPD > 2.5) [27]. Manley suggested that predictive models with an RPD higher than 8 were useful for any analytical application [28].

Furthermore, the relative standard deviations (RSD, %) were also calculated, thus reflecting the variability and robustness across folds.

RSD(%) = SD/mean

where SD is the standard deviation of 50 AUC (or accuracy or R^2^) values, and the mean is the mean of the 50 AUC (or accuracy or R^2^) values. The smaller the RSD%, the more stable the model.

The optimal models were determined according to the following rules: (1) Binary classification models: higher AUC, accuracy, sensitivity, specificity, PPV, and NPV; (2) multi-classification models: higher accuracy and kappa value; and (3) quantitative regression models: higher R^2^ and RPD and lower RMSE and MAE. When the above indicators of two models were equal, lower RSD was considered.

## 3. Results and Discussion

### 3.1. Quality Parameter Evaluation and FTIR Spectral Characteristics of the Milks

The chemical composition of cow milk, buffalo milk, goat milk, and camel milk determined by the MilkoScan FT+ (Foss, Hillerød, Denmark) instrument is shown in Table 3. Buffalo milk had the highest total solids, followed by camel milk and goat milk, while cow milk had the lowest. These data show the higher content of fat in buffalo milk, goat milk, and camel milk than in cow milk, and are in agreement with those reported in previous studies [6,18].

**Figure 1:**
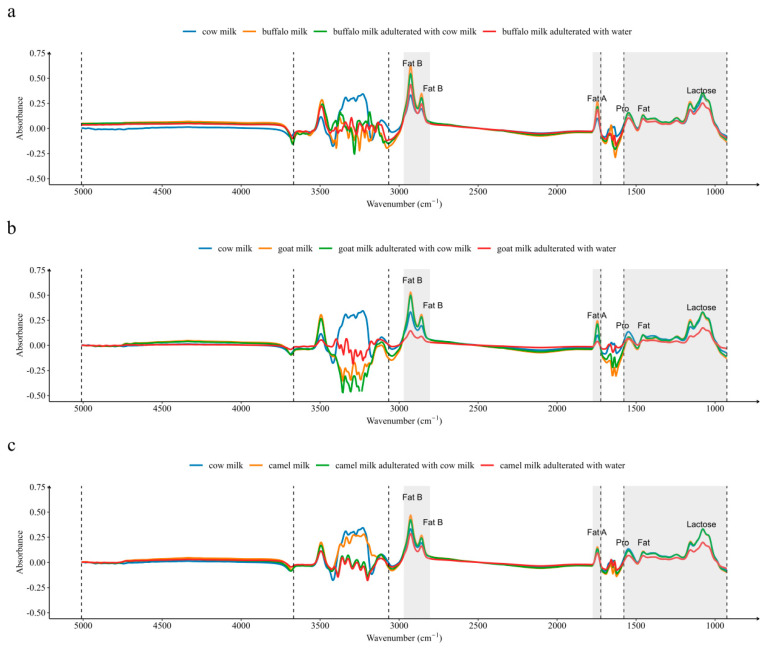
Original mid-infrared spectroscopy. (**a**) Buffalo milk; (**b**) Goat milk; (**c**) Camel milk. Gray shadow represents the region used for modeling.

According to the spectra, a region of approximately 2900 cm^−1^ provided information about the fat molecules [29], with buffalo milk having the highest peak, followed by goat milk and camel milk, with cow milk having the lowest peak. The regions at 1640 and 1540 cm^−1^ are associated with amide-I and amide-II, respectively, and provide information about the protein molecules [29]. In this region, buffalo milk has the highest peak. The absorption of goat milk in this region is lower than that of cow milk, which indicates that goat’s milk has lower protein levels than cow milk. The difference in absorbance between buffalo milk and cow milk was greater than that between goat milk, camel milk, and cow milk (Figure 1), corresponding to the results reported in the information on the composition of milk (Table 3).

As can be seen in Figure 1, the addition of water to pure milk leads to a decrease in the absorption peaks in each region. The change in absorbance after the addition of cow milk to pure milk is very small, except for the fat absorption region (decrease). Overall, there were visible differences in the spectra between pure milk and pure milk–water mixtures (buffalo, goat, and camel milk adulterated with cow milk), but there were similar spectra between pure milk and pure milk–cow milk mixtures. After removing the water region, bands of large differences were located at 2968 to 2802 cm^−1^ and 1773 to 1692 cm^−1^, and the absorbance in these bands is mainly related to fat content [30]. Other pronounced differences on spectra between pure milk and adulterated milk were located in the milk protein (1544 cm^−1^) and lactose (1159 and 1076 cm^−1^) absorption regions [7].

There were visible differences in spectra between pure milk and pure milk–water mixtures, and it may be possible to directly identify whether water was adulterated in buffalo milk, goat milk, and camel milk through spectrograms. However, the spectral differences between pure milk and pure milk–cow milk mixtures were so subtle that it was not possible to differentiate them by using a simple visual observation method. Furthermore, it is even more impossible to quantify the level of adulteration of cow milk. Nevertheless, with the help of machine learning algorithms, it may be possible to extract these differences by establishing a model for qualitative identification or quantitative analysis of its adulteration.

### 3.2. Models of Cow Milk or Water Adulteration in Buffalo Milk

As buffalo milk has a greater market demand, higher nutritional value, and is more commonly adulterated than goat milk and camel milk, this study focused on the specific process of modeling buffalo milk adulteration, and described it in detail. The nutritional value and flavor of buffalo milk is superior to that of cow milk, and the price is about twice that of cow milk [31]. The adulteration of buffalo milk with cow milk is a common fraud, which not only alters its nutritional and sensory properties, but also leads to cow milk intolerance or allergies. It is common to adulterate buffalo milk with at least 5% cow’s milk due to its profit [8], so in this study, we used 5% as the minimum level of adulteration. This is the first attempt to combine modern statistical machine learning algorithms with MIRS for identifying adulteration of cow milk or water in buffalo milk, goat milk, and camel milk. Overall, as expected, the ability of MIRS to predict water adulteration in buffalo milk was better than that of cow milk adulteration, because buffalo milk is more similar to cow milk than to water.

#### 3.2.1. Binary Classification Model for Identifying Buffalo–Cow Milk Mixtures or Buffalo Milk–Water Mixtures

Spectral preprocessing improved the model prediction quality, especially 1D and 2D preprocessing (Tables S1–S4), which displayed similar effects as the results of Soyeurt et al. [32]. Table 4 shows the performance of the binary classification model for identifying buffalo–cow milk mixtures and buffalo milk–water mixtures using three machine learning algorithms (PLSDA, LSVM, and RSVM).

The PLSDA was the optimal way to determine buffalo-cow milk mixtures, with the AUC, accuracy, sensitivity, specificity, PPV, and NPV of the validation sets all being one, and the RSD was 0.49%. The RSVM is the worst, with the AUC = 0.98, accuracy = 0.92, sensitivity = 0.95, specificity = 0.88, PPV = 0.89, and NPV = 0.95 in the validation set. Furthermore, RSD = 3.86%, indicating that the model stability was also worse than LSVM and PLSDA.

For the prediction of water adulteration in buffalo milk, the PLSDA also generated the optimal results, with the AUC, accuracy, sensitivity, specificity, PPV, and NPV all being one, and RSD = 0.55%, followed by LSVM and RSVM algorithms.

The evaluation of 15 models that were built based on three machine learning modeling algorithms and five MIRS preprocessing algorithms displayed that the binary classification model—using the 1D preprocessing algorithm and the PLSDA modeling algorithm—showed the highest prediction accuracy for identifying buffalo–cow milk mixtures. The model using the 2D preprocessing algorithm and the PLSDA modeling algorithm produced the highest prediction accuracy for identifying buffalo milk–water mixtures. These two models could roughly categorize buffalo milk into unadulterated (pure buffalo milk) and adulterated (buffalo milk–cow milk/water mixtures), with the AUC, accuracy, sensitivity, specificity, PPV, and NPV equal to one in the validation set.

**Figure 2:**
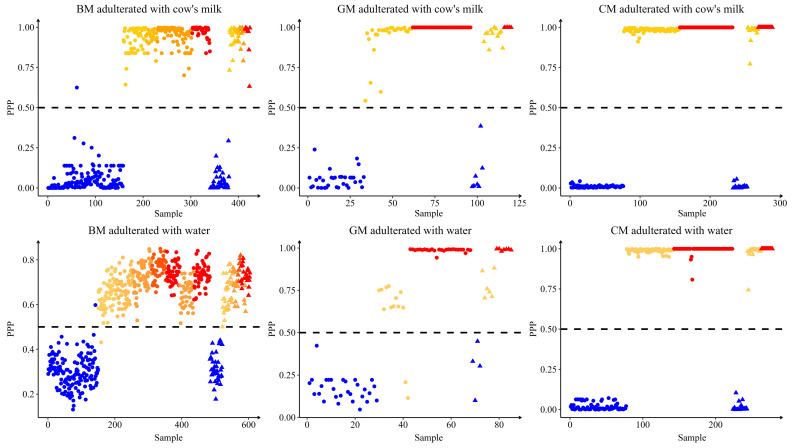
The discriminant results (positive predictive probability, PPP) of samples in calibration and validation sets using optimal models. BM = buffalo milk; GM = goat milk; CM = camel milk. Circles: calibration set; triangles: validation set; yellow, red and orange: adulterants (positive); blue: pure milk (negative). Shades of color indicate the level of adulteration.

Figure 2 illustrates the positive predictive probability (PPP) calculated by the binary optimal model. For individual milk samples, the prediction model can calculate PPP and negative prediction probability (NPP) that sum up to one. On the one hand, the model used this information to predict whether the data is positive (PPP > NPP) or negative (PPP < NPP). On the other hand, the probability can indicate the certainty of the model’s prediction, where a high probability suggests a high possibility of correct predictions [33]. It was found that for samples with a high adulteration proportion, PPP was also high (Figure 2), indicating that the model was more favorable in predicting samples with high adulteration. Interestingly, we also revealed that for samples with incorrect predictions, the PPP value given by the model was close to 0.50. This suggested that the certainty of making correct predictions for these sample models was poor. Therefore, to ensure that the predictions were unbiased, the predictive probability can also be considered for judging and identifying the samples. This study not only identifies the adulterated buffalo milk based on the results of the binary identification model (positive or negative), but it also does so with reference to the predictive probability of the model in order to improve the reliability of the model.

#### 3.2.2. Multi-Classification Models for Identifying High or Low Adulterant Level

Spectral preprocessing improved the model prediction ability (Tables S5 and S6). Table 5 shows the performance of the multi-classification model for identifying high or low adulterant levels (with a threshold of 25%) with cow milk or water in buffalo milk. The LSVM algorithm outperformed PLSDA, with a validation set accuracy of 0.94 and 0.99 for identifying the high or low proportion of cow milk or water adulteration in buffalo milk, respectively. In addition, a similar pattern to that reported by Silva et al. was found: the model presented a worse performance in buffalo milk with a low proportion of adulteration than for samples with a high proportion of adulteration (results not shown) [31].

The multi-classification model developed by LSVM modeling algorithm and the 1D spectral preprocessing algorithm for identifying the proportion of adulterated cow milk or water in buffalo milk showed the most favorable results, with an accuracy of 0.94 and 0.99, and kappa values of 0.90 and 0.99 in the validation set, respectively. The above two models can categorize buffalo milk as no adulteration, low level adulteration (adulteration proportion less than 25%), and high level adulteration (adulteration proportion more than 25%). Several studies have reported the identification of cow milk adulteration in buffalo milk based on MIRS. The accuracy of the binary classification model for identifying adulteration was 0.91 [10], and the accuracy of the multi-classification model for identifying the high and low levels of adulteration was 0.96 [31], which was similar to the results of the present study. Compared to these studies, this study showed several advantages: (1) the use of multiple modern statistical machine learning algorithms to develop a predictive model, and (2) the study of water adulteration, which is a very common form of adulteration.

#### 3.2.3. Quantitative Prediction of Adulteration in Buffalo Milk with Cow’s Milk and Water

MIRS can effectively and qualitatively identify adulterated buffalo milk. To further detect the level of adulteration accurately in buffalo milk, a regression modeling study was carried out to quantitatively predict the level of adulteration. Although it has been demonstrated that conventional machine learning algorithms (PLSR) can predict the level of cow milk in buffalo milk based on MIRS data [7,18,29,31,34], the aim of this study was to evaluate the predictive ability of modern machine learning algorithms and compare them with PLSR. Table 6 summarizes the performance of the regression models based on the 12 machine learning algorithms and the best MIRS preprocessing for predicting the proportion of cow milk and water adulteration in buffalo milk. Also, the predictive performance of the 12 machine learning algorithms using five MIRS preprocessing algorithms is shown in Tables S7 and S8. PLSR was considered a standard method because of its strong predictive performance in chemometric analyses. However, in the present study, PLSR did not show the best results.

For the regression model to quantify the proportion of cow milk adulteration in buffalo milk, LSVM, SSR, CART, RR, RF, and GBM all showed poor ability with a RPDV less than two. RSVM, PPR, BRNN, EN, and LASSO outperformed PLSR. BRNN had the best performance, and the RSD, which indicates the stability of the model, was the lowest among the 12 machine learning algorithms. Compared to the most commonly used PLSR, the BRNN reduced RMSEV and RSD by 1.54% and 4.62%, respectively, and improved R_V_^2^ and RPD_V_ by 0.09 and 0.53, respectively.

For the regression models quantifying the proportion of water adulteration in buffalo milk, all the models showed accurate and robust predictions, with R_V_^2^ higher than 0.95, and RPD_V_ higher than 5. LSVM, PPR, and BRNN outperformed PLSR, with a R_V_^2^ of 0.99 for all algorithms and RPD_V_ of 8.58, 11.30, 8.92, and 8.39, respectively. The PPR algorithm showed excellent predictive performance, with RMSEV = 1.67%, MAE_V_ = 0.59%, R_V_^2^ = 0.99, and RPD_V_ = 11.30. Compared to the PLSR algorithm, RMSEV was reduced by 0.58% and RPD_V_ was improved by 2.91 for PPR. BRNN displayed good predictive ability for any predictions. Studies have shown that NN can provide superior predictions compared to linear models [35,36]. However, NN are susceptible to overfitting, and may exhibit lower robustness in predicting new data. NN with Bayesian regularization (BR) training algorithms can avoid such overfitting.

The performance evaluation of 60 models based on 12 machine learning modeling algorithms and five MIRS preprocessing algorithms showed that BRNN and PPR were the optimal modeling algorithms to quantify the proportion of cow milk or water adulteration in buffalo milk, respectively. To further improve the performance of the models, based on the above two optimal models, PCA dimensionality reduction for MIRS data was conducted before modeling. That is, the principal components of 244 wavepoints after PCA were used for remodeling (PCA + BRNN and PCA + PPR), instead of using the 244 wavepoints for direct modeling (BRNN and PPR). Compared to BRNN and PPR, PCA+BRNN slightly improved the ability of MIRS to predict the proportion of cow milk adulteration in buffalo milk (RMSEV: 6.02% vs. 5.42%, R_V_^2^: 0.85 vs. 0.88, RPD_V_: 2.59 vs. 2.87), while PCA+PPR did not improve the ability of MIRS to predict the proportion of water adulteration in buffalo milk (RMSEV: 1.67% vs. 1.70%, R_V_^2^: 0.99 vs. 0.99, RPD_V_: 11.30 vs. 11.10). Although the models built by PCA+BRNN and PCA+PPR displayed similar accuracy to BRNN and PPR, the run times were considerably shorter, especially compared to the BRNN.

**Figure 3:**
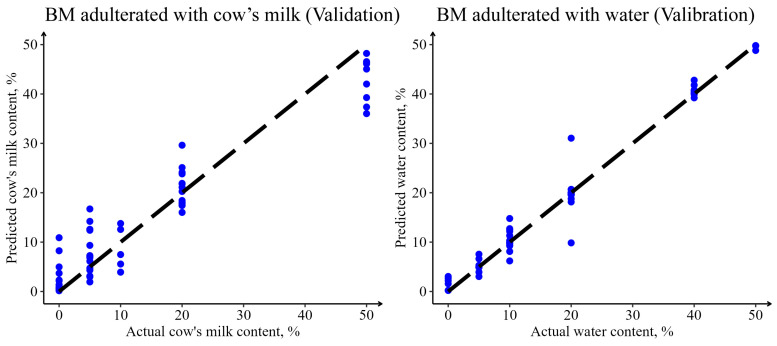
Observed vs. predicted levels (%) of adulterated cow’s milk or adulterated water content in buffalo milk using optimal models for the validation sets of samples.

Therefore, considering the model performance and time cost, it was found that the regression model based on the PCA+BRNN modeling algorithm and 1D preprocessing algorithm was the optimal way to predict the proportion of cow milk adulteration in buffalo milk, with RMSEV = 5.42%, MAE_V_ = 3.65%, R_V_^2^ = 0.88, and RPD_V_ = 2.87. The performance of a regression model based on the PCA + PPR modeling algorithm and 1D preprocessing algorithm was the optimal for predicting the proportion of water adulteration in buffalo milk, with RMSEV = 1.70%, MAE_V_ = 0.68%, R_V_^2^ = 0.99, and RPD_V_ = 11.10. The models developed above performed well (R^2^ > 0.85, RPD > 2.0), especially the model for predicting the proportion of water adulteration. These parameters indicated that the presented method had the potential to predict adulteration. For the water adulteration model, the method could be used for the routine analysis of milk samples for quality control, but for the cow milk adulteration model, the method was not adequate for routine applications. The relationship between the predicted adulteration proportion and true adulteration proportion is shown in Figure 3. In the regression model for predicting the proportion of water adulteration in buffalo milk, the relationship between predicted and true values followed y = x, even when the proportion of adulteration was as low as 5%. However, for the regression model predicting the proportion of cow milk adulteration in buffalo’s milk, the model predicted better only for samples where the proportion of adulteration was higher than 10%. Therefore, the developed MIRS model can accurately predict the proportion of cow milk adulteration in buffalo milk above 10% and the proportion of water adulteration above 5%; i.e., the model’s limit of quantitation (LOD) is 10% and 5%, respectively.

Various methods have been applied to detect cow’s milk adulteration in buffalo milk, such as the capillary electrophoresis technique [6], lateral flow immunoassay [37], liquid chromatography-tandem mass spectrometry [8], and frontal fluorescence spectroscopy [38], with an LOD in a range between 3.1% to 20%. The LOQ value (10%) of the present study is within this range and has a much lower prediction error. Moreover, the current method showed advantages in terms of rapidity, environmental impact, and high throughput measurement. Previous studies reported that the range of RMSE was from 0.23% to 7.42% by MIRS prediction of the proportion of cow’s milk adulteration in buffalo milk [3], which was similar to the results obtained in this study. Compared with other studies, our study presented more advantages, such as using the large amount of data and many modeling algorithms, as well as spectral preprocessing methods, which enabled the useful information contained in MIRS to be fully explored, and allowed robust prediction models to be developed.

### 3.3. Models of Cow Milk or Water Adulteration in Goat and Camel Milk

The production level of goat milk and camel milk is much lower than that of cow milk. It was reported that goat milk is very rich in terms of calcium, phosphorus, magnesium, copper, conjugated linoleic, and omega 3 and 6 fatty acids [7]. Camel milk is rich in potassium, sodium, copper, iron, magnesium, zinc, vitamins, insulin, and various bioactive substances [14]. Goat milk and camel is less allergenic than cow’s milk, so the addition of cow milk to these milks can have adverse health effects on people who have allergies and/or sensitive digestive systems [7]. Using the strategies and techniques for developing buffalo milk adulteration, we further developed models for identifying the adulteration of cow milk or water in goat milk and camel milk (Table 3 and Table 4). In this study, only two preliminary adulteration percentages, 20% and 50%, were set up, and only qualitative identification analyses were performed. LSVM was always the optimal and most robust modeling algorithm.

#### 3.3.1. Classification Model for Identifying Goat Milk Adulterated with Cow Milk or Water and Its Level of Adulteration (High or Low)

The binary classification model for identifying goat–cow milk mixtures or goat milk–water mixtures using the 1D preprocessing algorithm and LSVM modeling algorithm presented a high prediction accuracy, with AUC, Accuracy, Sensitivity, Specificity, PPV, and NPV equal to 1 in the validation set.

The multi-classification model for identifying low or high proportions of cow milk in goat milk using the LSVM modeling algorithm and 2D spectral preprocessing algorithm performed the best, with an accuracy of 1 and kappa value of 1 in the validation set. When the LSVM modeling algorithm and raw spectra were implemented, the multi-classification model for identifying the proportion of water adulteration in goat milk was optimal, with an accuracy and kappa value of 1 in the validation set.

Sen et al. used FTIR spectroscopy and orthogonal partial least squares discriminant analysis (OPLS-DA) to identify goat–cow milk mixtures, with a classification accuracy of 0.93 [7]. Differences in prediction accuracy were mainly attributed to adulteration concentrations, modeling algorithms, and spectral preprocessing methods.

#### 3.3.2. Classification Model for Identifying Camel Milk Adulterated with Cow Milk or Water and Its Level of Adulteration (High or Low)

The model for identifying camel milk adulteration showed superior performance than the model for goat milk, which mainly reflects on its robustness; i.e., lower RSD. The binary classification model for identifying camel–cow milk mixtures using 1D or 2D preprocessing algorithms and the LSVM modeling algorithm produced the highest predictive accuracy, and the binary classification model for identifying camel milk–water mixtures using 1D preprocessing algorithm and the PLSDA modeling algorithm or 2D preprocessing and the LSVM modeling algorithms displayed the highest predictive accuracy, with the AUC, accuracy, sensitivity, specificity, PPV, and NPV equal to 1.00, as well as RSD = 0% in both calibration and validation sets.

The multi-classification model for identifying the high or low proportion of adulterated cow’s milk in camel milk exhibited a favorable performance when the LSVM modeling algorithm and 1D spectral preprocessing algorithm were used. In addition, the LSVM and 2D algorithms-based multi-classification model displayed the best performance for identifying the high or low proportion of adulterated water in camel milk. These multi-classification models could achieve 100% of correct classifications.

The practicality of implementing these methods in different geographic regions and production scales of the dairy industry has yet to be considered, as the amount of data used in the modeling process of this paper is not sufficient for production applications (particularly regarding goat and camel milk models), and the data are only from one province in China, without external validation with data from other provinces. However, from the resultant parameters of the preliminary models obtained in this research, it is very promising that the developed models can be applied in the dairy industry in different geographic regions and at different production scales.

## 4. Conclusions

This study demonstrated that modern statistical machine learning algorithms (e.g., SVM, BRNN, and PPR) can improve the ability of MIRS to predict adulteration in buffalo milk, goat milk, and camel milk. We developed raw milk adulteration detection models based on MIRS and modern statistical machine learning algorithms, which can be used to detect fraudulent cow milk and water adulteration in buffalo milk, goat milk, and camel milk, with high classification accuracy (almost close to 1), prediction errors of 5.42% and 1.70%, and an LOQ of 10% and 5% (vol/vol). The SVM performed better in the classification model, and the BRNN performed better in the quantitative model; these two algorithms were recommended to be applied in other related research. This research is critical to tracking the quality of sold products, helping to ensure consumer confidence and market integrity. Further research efforts are recommended to collect samples from different regions and breeds in order to build more robust and accurate prediction models. In addition, attempts could be made to incorporate multiple adulterants in milk at the same time, which may be more consistent with production practices.

## Figures and Tables

**Table 1:** Sample number for each adulteration proportion.

Type	Proportion of Adulteration of Cow Milk (vol/vol) ^1^	n	Proportion of Adulteration of Water (vol/vol) ^1^	n
Buffalo milk	0%	198	0%	187
	5%	59	5%	68
	10%	34	10%	67
	20%	82	20%	119
	50%	49	40%	52
			50%	111
Goat milk	0%	40	0%	33
	20%	40	20%	19
	50%	40	50%	33
Camel milk	0%	97	0%	97
	20%	95	20%	84
	50%	70	50%	97

^1^ 10% means pure buffalo milk, goat milk or camel milk.

**Table 2:** Sample number for calibration and validation sets.

Model Type	Pure Milk–Adulterants	Calibration Sets	Validation Sets
Binary classification models	Buffalo milk–cow milk	338 (158 pure and 180 mixtures)	84 (40 pure and 44 mixtures)
	Buffalo milk–water	484 (150 pure and 334 mixtures)	120 (37 pure and 83 mixtures)
	Goat milk–cow milk	96 (33 pure and 63 mixtures)	24 (7 pure and 17 mixtures)
	Goat milk–water	68 (29 pure and 39 mixtures)	17 (4 pure and 13 mixtures)
	Camel milk–cow milk	210 (78 pure and 132 mixtures)	52 (19 pure and 33 mixtures)
	Camel milk–water	223 (77 pure and 146 mixtures)	55 (20 pure and 35 mixtures)
Multi-classification models	Buffalo milk–cow milk	338 (158 pure, 140 low, and 40 high)	84 (40 pure, 35 low, and 9 high)
	Buffalo milk–water	484 (141 pure, 212 low, and 131 high)	120 (46 pure, 42 low, and 32 high)
	Goat milk–cow milk	96 (32 pure, 32 low, and 32 high)	24 (8 pure, 8 low, and 8 high)
	Goat milk–water	69 (25 pure, 17 low, and 27 high)	16 (8 pure, 2 low, and 6 high)
	Camel milk–cow milk	210 (78 pure, 76 low, and 56 high)	52 (19 pure, 19 low, and 14 high)
	Camel milk–water	223 (84 pure, 61 low, and 78 high)	55 (13 pure, 23 low, and 19 high)
Quantitative regression models	Buffalo milk–cow milk	339	83
	Buffalo milk–water	484	120

**Table 3:** Mean ± standard deviation of the chemical composition of the milk samples.

Traits	Cow Milk	Buffalo Milk	Goat Milk	Camel Milk
Fat, %	3.54 ± 1.00 ^c^	7.76 ± 1.79 ^a^	5.39 ± 4.10 ^b^	5.52 ± 0.48 ^b^
Protein, %	3.59 ± 0.31 ^b^	4.83 ± 1.21 ^a^	3.29 ± 0.42 ^c^	3.71 ± 0.26 ^b^
Lactose, %	4.89 ± 0.31 ^bc^	4.97 ± 0.53 ^b^	4.77 ± 0.45 ^c^	5.11 ± 0.18 ^a^
SNF, %	9.10 ± 0.46 ^c^	10.72 ± 1.16 ^a^	8.58 ± 0.81 ^d^	9.59 ± 0.34 ^b^
TS, %	12.67 ± 1.22 ^d^	18.21 ± 2.38 ^a^	14.27 ± 4.61 ^c^	15.29 ± 0.72 ^b^
MUN, mg/100 g	10.52 ± 1.80 ^c^	12.11 ± 9.14 ^c^	31.95 ± 0.45 ^b^	39.43 ± 2.42 ^a^

Means within a row with different superscripts differ (*p* < 0.05).

**Table 4:** Performance of prediction models obtained in the calibration and validation sets, for the discrimination of pure milk and adulterant (mixed with milk or water) ^1^.

Milk Product ^2^	Preprocessing ^3^	Best Method ^4^	RSD, % ^5^	Calibration ^5^						Validation ^5^					
				AUC	Acc	Sen	Spe	PPV	NPV	AUC	Acc	Sen	Spe	PPV	NPV
adulterated with cow milk															
BM	1D	PLSDA	0.49	1.00	1.00	1.00	0.99	0.99	1.00	1.00	1.00	1.00	1.00	1.00	1.00
	1D	LSVM	1.94	1.00	1.00	1.00	0.99	0.99	1.00	1.00	1.00	1.00	1.00	1.00	1.00
	2D	RSVM	3.86	1.00	0.99	0.99	0.99	0.99	0.99	0.98	0.92	0.95	0.88	0.89	0.95
GM	2D	PLSDA	2.47	1.00	0.99	0.98	1.00	1.00	0.97	0.99	0.96	0.94	1.00	1.00	0.86
	1D	LSVM	3.54	1.00	1.00	1.00	1.00	1.00	1.00	1.00	1.00	1.00	1.00	1.00	1.00
	2D	RSVM	10.61	1.00	0.99	0.98	1.00	1.00	0.97	0.98	0.92	0.94	0.86	0.94	0.86
CM	2D	PLSDA	0.24	1.00	0.99	0.98	1.00	1.00	0.96	1.00	1.00	1.00	1.00	1.00	1.00
	1D/2D	LSVM	0.00	1.00	1.00	1.00	1.00	1.00	1.00	1.00	1.00	1.00	1.00	1.00	1.00
	2D	RSVM	0.98	1.00	1.00	1.00	1.00	1.00	1.00	0.99	0.95	0.97	0.90	0.95	0.95
adulterated with water															
BM	2D	PLSDA	0.55	1.00	1.00	1.00	0.99	1.00	0.99	1.00	1.00	1.00	1.00	1.00	1.00
	1D	LSVM	1.03	1.00	1.00	1.00	0.99	0.99	1.00	1.00	0.99	0.99	1.00	1.00	0.97
	1D	RSVM	0.74	1.00	1.00	1.00	1.00	1.00	1.00	1.00	0.98	0.98	0.97	0.99	0.95
GM	SNV	PLSDA	6.84	1.00	0.97	0.95	1.00	1.00	0.94	1.00	0.94	0.92	1.00	1.00	0.80
	1D	LSVM	9.58	0.97	0.97	0.95	1.00	1.00	0.94	1.00	1.00	1.00	1.00	1.00	1.00
	1D	RSVM	10.95	1.00	0.97	0.95	1.00	1.00	0.94	1.00	0.94	0.92	1.00	1.00	0.80
CM	1D	PLSDA	0.00	1.00	1.00	1.00	1.00	1.00	1.00	1.00	1.00	1.00	1.00	1.00	1.00
	2D	LSVM	0.00	1.00	1.00	1.00	1.00	1.00	1.00	1.00	1.00	1.00	1.00	1.00	1.00
	2D	RSVM	0.35	1.00	1.00	1.00	1.00	1.00	1.00	1.00	1.00	1.00	1.00	1.00	1.00

^1^ From the records, the positive cases were adulterant and negative cases were pure milk. ^2^ BM= buffalo milk; GM = goat milk; CM = camel milk. ^3^ 1D = first-order derivative, 2D = second-order derivative, SNV = standard normal variate. ^4^ PLS-DA = partial least squares discriminant analysis; LSVM = svmLinear (support vector machine with kernel); RSVM = svmRadial (support vector machine with radial basis function kernel). ^5^ Acc = Accuracy; Sen = Sensitivity; Spe = Specificity; PPV = positive predicted value; NPV = negative predicted value; AUC = area under the receiver operating characteristic curve; RSD = the relative standard deviations.

**Table 5:** Performance of prediction models obtained in the calibration and validation set, for the discrimination of no adulteration, low level adulteration (adulteration proportion less than 25%), and high level adulteration (adulteration proportion more than 25%) samples.

Type ^1^	Model ^2^	Adulterated with Cow’s Milk						Adulterated with Water					
		Preprocessing ^3^	RSD, % ^4^	Acc_c_	Kappa_c_	Acc_v_	Kappa_v_	Preprocessing ^3^	RSD, % ^4^	Acc_v_	Kappa_v_	Acc_v_	Kappa_v_
BM	PLSDA	1D	3.39	0.97	0.96	0.91	0.84	1D	2.60	0.98	0.97	0.96	0.94
	LSVM	1D	3.49	0.99	0.98	0.94	0.90	1D	1.02	1.00	1.00	0.99	0.99
	RSVM	2D	6.63	0.96	0.94	0.83	0.71	1D	1.97	1.00	1.00	0.99	0.99
GM	PLSDA	None	12.64	0.99	0.98	0.96	0.94	1D	18.17	1.00	1.00	0.94	0.90
	LSVM	2D	7.06	1.00	1.00	1.00	1.00	None	9.54	0.97	0.96	1.00	1.00
	RSVM	2D	17.97	0.99	0.98	0.92	0.88	2D	19.81	0.96	0.93	1.00	1.00
CM	PLSDA	1D	5.39	0.99	0.98	1.00	1.00	1D	1.51	1.00	1.00	1.00	1.00
	LSVM	1D	0.00	1.00	1.00	1.00	1.00	2D	1.77	1.00	1.00	1.00	1.00
	RSVM	1D	5.12	1.00	1.00	0.98	0.97	2D	2.19	1.00	1.00	1.00	1.00

^1^ BM = buffalo milk; GM = goat milk; CM = camel milk; ^2^ PLS-DA = partial least squares discriminant analysis; LSVM = svmLinear (support vector machine with kernel); RSVM = svmRadial (support vector machine with radial basis function kernel). ^3^ 1D = first-order derivative, 2D = second-order derivative. ^4^ Acc_c_ = accuracy in calibration set, kappa_c_ = kappa value in calibration set, Acc_v_ = accuracy in validation set, kappa_v_ = kappa value in validation set, and RSD = the relative standard deviations.

**Table 6:** Comparison of the partial least squares regression (PLS), and 12 modern statistical machine learning algorithms of the Fourier transform infrared spectra for determining the adulterated cow milk or water level in buffalo milk.

Modeling ^2^	Pre ^3^	RSD ^1^	Calibration Set ^1^				Validation Set ^1^			
			RMSEC	MAE_C_	R_C_^2^	RPD_C_	RMSEV	MAE_V_	R_V_^2^	RPD_V_
adulterated with cow’s milk										
PLSR	SNV	10.18	6.53	4.89	0.83	2.46	7.56	5.83	0.76	2.06
LSVM	SNV	9.41	6.68	4.66	0.84	2.40	7.88	5.87	0.74	1.98
RSVM	2D	9.36	4.60	2.51	0.92	3.49	7.21	5.12	0.79	2.16
SSR	SNV	12.32	8.33	6.27	0.74	1.93	8.22	6.38	0.72	1.89
PPR	None	10.15	2.75	1.23	0.97	5.84	7.37	3.73	0.77	2.11
CART	SG	40.40	8.21	4.90	0.74	1.95	11.51	6.80	0.49	1.35
BRNN	SG	5.56	4.94	3.27	0.91	3.25	6.02	4.09	0.85	2.59
RR	SNV	11.06	8.24	6.23	0.75	1.95	8.28	6.46	0.71	1.88
EN	SNV	8.47	6.41	4.90	0.84	2.50	7.45	5.69	0.77	2.09
LASSO	SNV	7.84	6.32	4.83	0.84	2.54	7.36	5.63	0.77	2.12
RF	2D	12.69	3.77	2.35	0.96	4.25	8.25	5.70	0.72	1.89
GBM	1D	14.19	4.15	2.82	0.93	3.87	8.81	6.01	0.70	1.77
PCA+BRNN	1D	6.71	5.74	3.88	0.87	2.80	5.42	3.65	0.88	2.87
adulterated with water										
PLSR	None	1.29	2.06	1.31	0.99	9.19	2.25	1.50	0.99	8.39
LSVM	1D	1.28	2.09	1.32	0.99	9.08	2.20	1.48	0.99	8.58
RSVM	1D	1.25	1.66	1.29	0.99	11.40	2.76	1.86	0.98	6.85
SSR	1D	1.37	2.39	1.50	0.98	7.91	2.47	1.73	0.98	7.64
PPR	SG	1.32	1.03	0.30	1.00	18.30	1.67	0.59	0.99	11.30
CART	2D	3.16	1.91	0.57	0.99	9.90	3.23	1.09	0.97	5.86
BRNN	SG	1.40	1.80	1.15	0.99	10.52	2.12	1.32	0.99	8.92
RR	1D	1.43	2.61	1.71	0.98	7.25	2.58	1.86	0.98	7.33
EN	1D	1.36	2.24	1.43	0.99	8.46	2.37	1.66	0.98	7.97
LASSO	1D	1.36	2.43	1.54	0.98	7.79	2.50	1.76	0.98	7.57
RF	2D	1.33	1.10	0.52	1.00	17.26	2.64	1.41	0.98	7.17
GBM	1D	0.93	0.18	0.07	1.00	104.93	2.51	1.35	0.98	7.53
PCA+PPR	1D	1.27	1.23	0.44	1.00	15.40	1.70	0.68	0.99	11.10

^1^ RSD = the relative standard deviations, RMSEC = root mean square error in calibration set, RMSEV = root mean square error in validation set, MAE_C_ = the mean absolute error in calibration set, MAE_V_ = the mean absolute error in validation set, R_C_^2^ = the coefficient of determination in calibration set, R_V_^2^ = the coefficient of determination in validation set, and RPD = the ratio performance deviation. ^2^ PLSR = partial least squares regression, LSVM = svmLinear (support vector machine with kernel), RSVM = svmRadial (support vector machine with radial basis function kernel), SSR = spike and slab regression, PPR = projection pursuit regression, CART = classification and regression tree, BRNN = bayesian regularized neural networks, RR = ridge regression, EN = elastic net regression, LASSO = least absolute shrinkage and selection operator; RF = random forest, and GBM = gradient boosting machine. ^3^ SG = Savitzky–Golay convolution smoothing, 1D = first-order derivative, 2D = second-order derivative, and SNV = standard normal variate.

## Data Availability

The data presented in this study are available on request from the corresponding author.

## Supplementary Materials

The following supporting information can be downloaded at: https://www.mdpi.com/article/10.3390/foods12203856/s1, Table S1: Performance of prediction models obtained in calibration set, for the discrimination of pure milk and adulterant (mixed with cow milk); Table S2: Performance of prediction models obtained in validation set, for the discrimination of pure milk and adulterant (mixed with cow milk); Table S3: Performance of prediction models obtained in calibration set, for the discrimination of pure milk and adulterant (mixed with water); Table S4: Performance of prediction models obtained in validation set, for the discrimination of pure milk and adulterant (mixed with water); Table S5: Performance of prediction models obtained in calibration and validation set, for the discrimination of no adulteration, low level adulteration (cow milk adulteration proportion less than 25%), and high level adulteration (cow milk adulteration proportion more than 25%) samples; Table S6: Performance of prediction models obtained in calibration and validation set, for the discrimination of no adulteration, low level adulteration (water adulteration proportion less than 25%), and high level adulteration (water adulteration proportion more than 25%) samples; Table S7: Comparison of the partial least squares (PLS) regression, and 11 modern statistical machine learning algorithms of the Fourier transform infrared spectra for determining the adulterated cow milk level in buffalo milk; Table S8: Comparison of the partial least squares (PLS) regression, and 11 modern statistical machine learning algorithms of the Fourier transform infrared spectra for determining the adulterated water level in buffalo milk.
