# Supplementary material for: Possible Alternatives: Identifying and Quantifying Adulteration in Buffalo, Goat, and Camel Milk Using Mid-Infrared Spectroscopy Combined with Modern Statistical Machine Learning Methods
Source: Foods. 2023 Oct 21;12(20):3856. doi: 10.3390/foods12203856 (PMC10606090; doi:10.3390/foods12203856)
Supplement: Supplementary file 1 [file foods-12-03856-s001.zip › foods-2650779-supplementary.pdf]

Table S1 Performance of prediction models obtained in calibration set, for the discrimination of pure milk and adulterant (mixed with cow milk)

Modeling[1]	Milk product[2]	Preprocessing[3]	Calibration Metrics[4]					
			**AUC**	**Acc**	**Sen**	**Spe**	**PPV**	**NPV**
PLSDA	BM	None	1.00	0.98	0.99	0.97	0.97	0.99
		**1D**	**1.00**	**1.00**	**1.00**	**0.99**	**0.99**	**1.00**
		**2D**	**1.00**	**1.00**	**1.00**	**0.99**	**0.99**	**1.00**
		SNV	1.00	0.99	0.99	0.99	0.99	0.99
		SG	1.00	0.98	1	0.96	0.97	1.00
	CM	None	1.00	1.00	0.99	1.00	1.00	0.99
		1D	1.00	1.00	0.99	1.00	1.00	0.99
		**2D**	**1.00**	**0.99**	**0.98**	**1.00**	**1.00**	**0.96**
		SNV	1.00	1.00	0.99	1.00	1.00	0.99
		SG	1.00	0.99	0.99	1.00	1.00	0.97
	GM	None	1.00	1.00	1.00	1.00	1.00	1.00
		1D	1.00	1.00	1.00	1.00	1.00	1.00
		**2D**	**1.00**	**0.99**	**0.98**	**1.00**	**1.00**	**0.97**
		SNV	1.00	1.00	1.00	1.00	1.00	1.00
		SG	1.00	1.00	1.00	1.00	1.00	1.00
LSVM	BM	None	1.00	0.99	0.99	0.98	0.98	0.99
		**1D**	**1.00**	**1.00**	**1.00**	**0.99**	**0.99**	**1.00**
		2D	1.00	1.00	1.00	0.99	0.99	1.00
		SNV	1.00	1.00	1.00	0.99	0.99	1.00
		SG	1.00	0.99	0.99	0.99	0.99	0.99
	CM	None	1.00	1.00	1.00	1.00	1.00	1.00
		**1D**	**1.00**	**1.00**	**1.00**	**1.00**	**1.00**	**1.00**
		**2D**	**1.00**	**1.00**	**1.00**	**1.00**	**1.00**	**1.00**
		SNV	1.00	0.99	0.99	1.00	1.00	0.99
		SG	1.00	1.00	1.00	1.00	1.00	1.00
	GM	None	1.00	0.99	0.98	1.00	1.00	0.97
		**1D**	**1.00**	**1.00**	**1.00**	**1.00**	**1.00**	**1.00**
		2D	1.00	0.98	0.97	1.00	1.00	0.94
		SNV	0.98	0.95	0.95	0.94	0.97	0.91
		SG	0.99	0.98	0.97	1.00	1.00	0.94
RSVM	BM	None	0.98	0.94	0.93	0.94	0.95	0.93
		1D	1.00	0.98	0.98	0.97	0.98	0.98
		**2D**	**1.00**	**0.99**	**0.99**	**0.99**	**0.99**	**0.99**
		SNV	1.00	1.00	1.00	1.00	1.00	1.00
		SG	0.98	0.93	0.92	0.94	0.95	0.91
	CM	**None**	**1.00**	**1.00**	**1.00**	**1.00**	**1.00**	**1.00**
		**1D**	**1.00**	**1.00**	**1.00**	**1.00**	**1.00**	**1.00**
		**2D**	**1.00**	**1.00**	**1.00**	**1.00**	**1.00**	**1.00**

		**SNV**	**1.00**	**1.00**	**1.00**	**1.00**	**1.00**	**1.00**
		SG	1.00	1.00	1.00	1.00	1.00	1.00
	GM	None	0.98	0.92	0.92	0.91	0.95	0.86
		1D	0.99	0.94	0.92	0.97	0.98	0.87
		**2D**	**1.00**	**0.99**	**0.98**	**1.00**	**1.00**	**0.97**
		SNV	0.99	0.94	0.95	0.91	0.95	0.91
		SG	0.98	0.91	0.92	0.88	0.94	0.85

[1]PLS-DA = partial least squares discriminant analysis; LSVM=svmLinear(support vector machine with kernel); RSVM=svmRadial(support vector machine with radial basis function kernel).
[2]BM= buffola milk; GM= goat milk; CM= camel milk.
[3]1D = first-order derivative , 2D = second-order derivative , SNV = Standard Normal Variate, and SG = Savitzky–Golsy convolution smoothing.
[4]Acc= Accuarcy; Sen=Sensitivity; Spe=Specificity; PPV = positive predicted value; NPV = negative predicted value; AUC = area under the receiver operating characteristic curve.

Table S2 Performance of prediction models obtained in validation set, for the discrimination of pure milk and adulterant (mixed with cow milk)

**Modeling[1]**	**Milk product[2]**	**Preprocessing[3]**	**Validation Metrics[4]**					
			**AUC**	**Acc**	**Sen**	**Spe**	**PPV**	**NPV**
PLSDA	BM	None	1.00	1.00	1.00	1.00	1.00	1.00
		**1D**	**1.00**	**1.00**	**1.00**	**1.00**	**1.00**	**1.00**
		**2D**	**1.00**	**1.00**	**1.00**	**1.00**	**1.00**	**1.00**
		SNV	1.00	1.00	1.00	1.00	1.00	1.00
		SG	1.00	1.00	1.00	1.00	1.00	1.00
	CM	None	1.00	0.96	0.94	1.00	1.00	0.91
		1D	1.00	0.98	0.97	1.00	1.00	0.95
		**2D**	**1.00**	**1.00**	**1.00**	**1.00**	**1.00**	**1.00**
		SNV	1.00	0.98	0.97	1.00	1.00	0.95
		SG	1.00	0.98	0.97	1.00	1.00	0.95
	GM	None	0.87	0.96	1.00	0.86	0.94	1.00
		1D	0.94	0.96	1.00	0.86	0.94	1.00
		**2D**	**0.99**	**0.96**	**0.94**	**1.00**	**1.00**	**0.86**
		SNV	0.95	0.92	0.94	0.86	0.94	0.86
		SG	0.95	0.96	1.00	0.86	0.94	1.00
LSVM	BM	None	1.00	0.98	1.00	0.95	0.96	1.00
		**1D**	**1.00**	**1.00**	**1.00**	**1.00**	**1.00**	**1.00**
		2D	1.00	0.99	0.98	1.00	1.00	0.98
		SNV	1.00	0.99	1.00	0.98	0.98	1.00
		SG	1.00	0.98	1.00	0.95	0.96	1.00
	CM	None	1.00	0.98	1.00	0.95	0.97	1.00
		**1D**	**1.00**	**1.00**	**1.00**	**1.00**	**1.00**	**1.00**

		**2D**	**1.00**	**1.00**	**1.00**	**1.00**	**1.00**	**1.00**
		SNV	0.99	0.98	0.97	1.00	1.00	0.95
		SG	0.99	0.95	0.97	0.90	0.95	0.95
	GM	None	1.00	0.96	0.94	1.00	1.00	0.88
		**1D**	**1.00**	**1.00**	**1.00**	**1.00**	**1.00**	**1.00**
		2D	1.00	1.00	1.00	1.00	1.00	1.00
		SNV	0.93	0.79	0.77	0.86	0.93	0.60
		SG	1.00	0.83	0.77	1.00	1.00	0.64
RSVM	BM	None	0.94	0.86	0.91	0.80	0.83	0.89
		1D	0.97	0.89	0.95	0.83	0.86	0.94
		**2D**	**0.98**	**0.92**	**0.95**	**0.88**	**0.89**	**0.95**
		SNV	0.90	0.81	0.77	0.85	0.85	0.77
		SG	0.94	0.86	0.91	0.80	0.83	0.89
	CM	**None**	**0.99**	**0.95**	**0.97**	**0.90**	**0.95**	**0.95**
		**1D**	**0.99**	**0.95**	**0.97**	**0.90**	**0.95**	**0.95**
		**2D**	**0.99**	**0.95**	**0.97**	**0.90**	**0.95**	**0.95**
		**SNV**	**0.99**	**0.95**	**0.97**	**0.90**	**0.95**	**0.95**
		SG	0.98	0.93	0.97	0.86	0.92	0.95
	GM	None	0.71	0.71	0.71	0.71	0.86	0.50
		1D	0.82	0.67	0.65	0.71	0.85	0.45
		**2D**	**0.98**	**0.92**	**0.94**	**0.86**	**0.94**	**0.86**
		SNV	0.83	0.71	0.71	0.71	0.86	0.50
		SG	0.71	0.71	0.71	0.71	0.86	0.50

[1]PLS-DA = partial least squares discriminant analysis; LSVM=svmLinear(support vector machine with kernel); RSVM=svmRadial(support vector machine with radial basis function kernel).

[2]BM= buffola milk; GM= goat milk; CM= camel milk.

[3]1D = first-order derivative , 2D = second-order derivative , SNV = Standard Normal Variate, and SG = Savitzky–Golsy convolution smoothing.

[4]Acc= Accuarcy; Sen=Sensitivity; Spe=Specificity; PPV = positive predicted value; NPV = negative predicted value; AUC = area under the receiver operating characteristic curve.

Table S3 Performance of prediction models obtained in calibration set, for the discrimination of pure milk and adulterant (mixed with water)

**Modeling[1]**	**Milk product[2]**	**Preprocessing[3]**	**Calibration Metrics[4]**					
			**AUC**	**Acc**	**Sen**	**Spe**	**PPV**	**NPV**
PLSDA	BM	None	1.00	0.99	0.99	0.99	0.99	0.98
		1D	1.00	0.99	0.99	0.99	1.00	0.99
		**2D**	**1.00**	**1.00**	**1.00**	**0.99**	**1.00**	**0.99**
		SNV	1.00	0.99	0.98	0.99	1.00	0.96
		SG	1.00	0.99	0.99	0.98	0.99	0.97
	CM	None	1.00	1.00	0.99	1.00	1.00	0.99
		**1D**	**1.00**	**1.00**	**1.00**	**1.00**	**1.00**	**1.00**

		**2D**	**1.00**	**1.00**	**1.00**	**1.00**	**1.00**	**1.00**
		**SNV**	**1.00**	**1.00**	**1.00**	**1.00**	**1.00**	**1.00**
		SG	**1.00**	**1.00**	**1.00**	**1.00**	**1.00**	**1.00**
	GM	None	1.00	1.00	1.00	1.00	1.00	1.00
		1D	1.00	1.00	1.00	1.00	1.00	1.00
		2D	1.00	1.00	1.00	1.00	1.00	1.00
		**SNV**	**1.00**	**0.97**	**0.95**	**1.00**	**1.00**	**0.94**
		SG	1.00	1.00	1.00	1.00	1.00	1.00
LSVM	BM	None	1.00	0.99	1.00	0.98	0.99	0.99
		1D	**1.00**	**1.00**	**1.00**	**0.99**	**0.99**	**1.00**
		2D	1.00	1.00	1.00	0.99	0.99	1.00
		SNV	1.00	1.00	1.00	0.99	0.99	0.99
		SG	1.00	0.99	0.99	0.99	0.99	0.98
	**CM**	**None**	**1.00**	**1.00**	**1.00**	**1.00**	**1.00**	**1.00**
		**1D**	**1.00**	**1.00**	**1.00**	**1.00**	**1.00**	**1.00**
		**2D**	**1.00**	**1.00**	**1.00**	**1.00**	**1.00**	**1.00**
		**SNV**	**1.00**	**1.00**	**1.00**	**1.00**	**1.00**	**1.00**
		**SG**	**1.00**	**1.00**	**1.00**	**1.00**	**1.00**	**1.00**
	GM	None	1.00	0.97	0.95	1.00	1.00	0.94
		**1D**	**0.97**	**0.97**	**0.95**	**1.00**	**1.00**	**0.94**
		2D	0.96	0.97	0.95	1.00	1.00	0.94
		SNV	1.00	0.99	0.97	1.00	1.00	0.97
		SG	1.00	0.97	0.95	1.00	1.00	0.94
RSVM	BM	None	1.00	0.98	0.99	0.97	0.99	0.97
		**1D**	**1.00**	**1.00**	**1.00**	**1.00**	**1.00**	**1.00**
		2D	1.00	0.99	0.99	0.99	0.99	0.99
		SNV	1.00	1.00	1.00	1.00	1.00	1.00
		SG	0.98	0.93	0.92	0.94	0.95	0.91
	CM	None	1.00	1.00	1.00	1.00	1.00	1.00
		1D	1.00	0.99	0.99	1.00	1.00	0.99
		**2D**	**1.00**	**1.00**	**1.00**	**1.00**	**1.00**	**1.00**
		SNV	1.00	1.00	1.00	1.00	1.00	1.00
		SG	1.00	0.99	0.99	1.00	1.00	0.99
	GM	None	0.98	0.97	0.95	1.00	1.00	0.94
		**1D**	**1.00**	**0.97**	**0.95**	**1.00**	**1.00**	**0.94**
		2D	0.99	0.97	0.95	1.00	1.00	0.94
		SNV	0.97	0.90	0.87	0.93	0.94	0.84
		SG	0.98	0.97	0.95	1.00	1.00	0.94

[1]PLS-DA = partial least squares discriminant analysis; LSVM=svmLinear(support vector machine with kernel); RSVM=svmRadial(support vector machine with radial basis

function kernel).
[2]BM= buffola milk; GM= goat milk; CM= camel milk.
[3]1D = first-order derivative , 2D = second-order derivative , SNV = Standard Normal Variate, and SG = Savitzky–Golsy convolution smoothing.
[4]Acc= Accuarcy; Sen=Sensitivity; Spe=Specificity; PPV = positive predicted value; NPV = negative predicted value; AUC = area under the receiver operating characteristic curve.

Table S4 Performance of prediction models obtained in validation set, for the discrimination of pure milk and adulterant (mixed with water)

**Modeling[1]**	**Milk product[2]**	**Preprocessing[3]**	**Validation Metrics[4]**					
			**AUC**	**Acc**	**Sen**	**Spe**	**PPV**	**NPV**
PLSDA	BM	None	1.00	1.00	1.00	1.00	1.00	1.00
		1D	1.00	1.00	1.00	1.00	1.00	1.00
		**2D**	**1.00**	**1.00**	**1.00**	**1.00**	**1.00**	**1.00**
		SNV	1.00	0.97	0.98	0.95	0.98	0.95
		SG	1.00	1.00	1.00	1.00	1.00	1.00
	CM	None	1.00	1.00	1.00	1.00	1.00	1.00
		**1D**	**1.00**	**1.00**	**1.00**	**1.00**	**1.00**	**1.00**
		**2D**	**1.00**	**1.00**	**1.00**	**1.00**	**1.00**	**1.00**
		**SNV**	**1.00**	**1.00**	**1.00**	**1.00**	**1.00**	**1.00**
		SG	**1.00**	**1.00**	**1.00**	**1.00**	**1.00**	**1.00**
	GM	None	0.94	0.88	0.92	0.75	0.92	0.75
		1D	0.96	0.88	0.85	1.00	1.00	0.67
		2D	0.85	0.76	0.69	1.00	1.00	0.50
		**SNV**	**1.00**	**0.94**	**0.92**	**1.00**	**1.00**	**0.80**
		SG	0.94	0.88	0.85	1.00	1.00	0.67
LSVM	BM	None	1.00	0.98	0.98	0.97	0.99	0.97
		**1D**	**1.00**	**0.99**	**0.99**	**1.00**	**1.00**	**0.97**
		2D	1.00	0.98	0.98	0.97	0.99	0.97
		SNV	1.00	0.98	0.99	0.97	0.99	0.97
		SG	1.00	0.99	1.00	0.97	0.99	1.00
	CM	**None**	**1.00**	**1.00**	**1.00**	**1.00**	**1.00**	**1.00**
		**1D**	**1.00**	**1.00**	**1.00**	**1.00**	**1.00**	**1.00**
		**2D**	**1.00**	**1.00**	**1.00**	**1.00**	**1.00**	**1.00**
		**SNV**	**1.00**	**1.00**	**1.00**	**1.00**	**1.00**	**1.00**
		**SG**	**1.00**	**1.00**	**1.00**	**1.00**	**1.00**	**1.00**
	GM	None	1.00	0.94	0.92	1.00	1.00	0.80
		**1D**	**1.00**	**1.00**	**1.00**	**1.00**	**1.00**	**1.00**

		2D	1.00	1.00	1.00	1.00	1.00	1.00
		SNV	0.94	0.82	0.85	0.75	0.92	0.60
		SG	1.00	0.94	0.92	1.00	1.00	0.80
RSVM	BM	None	0.99	0.94	0.95	0.92	0.96	0.89
		**1D**	**1.00**	**0.98**	**0.98**	**0.97**	**0.99**	**0.95**
		2D	0.98	0.92	0.95	0.88	0.89	0.95
		SNV	0.90	0.81	0.77	0.85	0.85	0.77
		SG	0.94	0.86	0.91	0.80	0.88	0.89
	CM	None	1.00	0.87	1.00	0.65	0.83	1.00
		1D	1.00	1.00	1.00	1.00	1.00	1.00
		**2D**	**1.00**	**1.00**	**1.00**	**1.00**	**1.00**	**1.00**
		SNV	0.99	0.91	1.00	0.75	0.88	1.00
		SG	1.00	0.89	1.00	0.70	0.85	1.00
	GM	None	0.96	0.88	0.85	1.00	1.00	0.67
		**1D**	**1.00**	**0.94**	**0.92**	**1.00**	**1.00**	**0.80**
		2D	1.00	0.94	0.92	1.00	1.00	0.80
		SNV	0.44	0.53	0.62	0.25	0.73	0.17
		SG	0.98	0.88	0.85	1.00	1.00	0.67

[1]PLS-DA = partial least squares discriminant analysis; LSVM=svmLinear(support vector machine with kernel); RSVM=svmRadial(support vector machine with radial basis function kernel).

[2]BM= buffola milk; GM= goat milk; CM= camel milk.

[3]1D = first-order derivative , 2D = second-order derivative , SNV = Standard Normal Variate, and SG = Savitzky–Golsy convolution smoothing.

[4]Acc= Accuarcy; Sen=Sensitivity; Spe=Specificity; PPV = positive predicted value; NPV = negative predicted value; AUC = area under the receiver operating characteristic curve.

Table S5 Performance of prediction models obtained in calibration and validation set, for the discrimination of no adulteration, low level adulteration (cow milk adulteration proportion less than 25%) and high level adulteration (cow milk adulteration proportion more than 25%) samples.

**Modeling[1]**	**Milk product[2]**	**Preprocessing[3]**	**Calibration**		**Validation**	
			**Acc[4]**	**KAPPA**	**Acc[4]**	**KAPPA**
PLSDA	BM	None	0.98	0.96	0.88	0.81
		**1D**	**0.97**	**0.96**	**0.91**	**0.84**
		2D	0.98	0.96	0.91	0.84
		SNV	0.98	0.96	0.91	0.84
		SG	0.96	0.93	0.89	0.82
	CM	None	0.98	0.97	1	1

		**1D**	**0.99**	**0.98**	**1**	**1**
		2D	0.97	0.96	0.97	0.95
		SNV	0.95	0.93	0.97	0.95
		SG	0.98	0.97	1	1
	GM	**None**	**0.99**	**0.98**	**0.96**	**0.94**
		1D	0.99	0.98	0.88	0.81
		2D	0.99	0.99	0.92	0.88
		SNV	0.99	0.98	0.88	0.81
		SG	0.98	0.97	0.88	0.81
LSVM	BM	None	0.98	0.97	0.93	0.88
		**1D**	**0.99**	**0.98**	**0.94**	**0.90**
		2D	0.98	0.97	0.88	0.81
		SNV	0.98	0.97	0.88	0.81
		SG	0.97	0.96	0.89	0.82
	CM	**None**	**1.00**	**1.00**	**1.00**	**1.00**
		**1D**	**1.00**	**1.00**	**1.00**	**1.00**
		**2D**	**1.00**	**1.00**	**1.00**	**1.00**
		**SNV**	**1.00**	**1.00**	**1.00**	**1.00**
		**SG**	**1.00**	**1.00**	**1.00**	**1.00**
	GM	None	0.96	0.94	1.00	1.00
		1D	1.00	1.00	0.96	0.94
		**2D**	**1.00**	**1.00**	**1.00**	**1.00**
		SNV	0.98	0.97	0.96	0.94
		SG	0.96	0.94	0.96	0.94
RSVM	BM	None	0.92	0.87	0.79	0.64
		1D	0.96	0.93	0.77	0.62
		**2D**	**0.96**	**0.94**	**0.83**	**0.71**
		SNV	0.96	0.93	0.73	0.54
		SG	0.91	0.85	0.77	0.62
	CM	None	1.00	1.00	0.91	0.87
		**1D**	**1.00**	**1.00**	**0.98**	**0.97**
		**2D**	**1.00**	**1.00**	**0.98**	**0.97**
		SNV	1.00	1.00	0.96	0.95
		SG	1.00	1.00	0.93	0.89
	GM	None	0.82	0.73	0.63	0.44
		1D	0.95	0.92	0.75	0.63
		**2D**	**0.99**	**0.98**	**0.92**	**0.88**
		SNV	0.92	0.88	0.88	0.81
		SG	0.83	0.75	0.63	0.44

[1]PLS-DA = partial least squares discriminant analysis; LSVM=svmLinear(support vector machine with kernel); RSVM=svmRadial(support vector machine with radial basis function kernel).

[2]BM= buffola milk; GM= goat milk; CM= camel milk.

[3]1D = first-order derivative , 2D = second-order derivative , SNV = Standard Normal Variate, and SG = Savitzky–Golsy convolution smoothing.

[4]Acc= Accuarcy.

Table S6 Performance of prediction models obtained in calibration and validation set, for the discrimination of no adulteration, low level adulteration (water adulteration proportion less than 25%) and high level adulteration (water adulteration proportion more than 25%) samples.

**Modeling[1]**	**Milk product[2]**	**Preprocessing[3]**	**Calibration**		**Validation**	
			**Acc[4]**	**KAPPA**	**Acc[4]**	**KAPPA**
PLSDA	BM	None	0.98	0.97	0.94	0.91
		**1D**	**0.98**	**0.97**	**0.96**	**0.94**
		2D	0.98	0.97	0.94	0.91
		SNV	0.97	0.95	0.94	0.91
		SG	0.96	0.93	0.93	0.89
	CM	None	1	1	1	1
		**1D**	**1**	**1**	**1**	**1**
		2D	1	1	1	1
		SNV	1	1	0.98	0.97
		SG	1	1	1	1
	GM	None	0.99	0.98	0.94	0.90
		**1D**	**1**	**1**	**0.94**	**0.90**
		2D	1	1	0.94	0.90
		SNV	0.99	0.98	0.75	0.58
		SG	1.00	1.00	0.94	0.90
LSVM	BM	None	0.99	0.99	0.98	0.97
		**1D**	**1.00**	**1.00**	**0.99**	**0.99**
		2D	1.00	1.00	0.98	0.96
		SNV	1.00	1.00	0.98	0.96
		SG	0.99	0.99	0.98	0.97
	CM	None	0.99	0.98	1.00	1.00
		1D	0.99	0.98	1.00	1.00
		**2D**	**1.00**	**1.00**	**1.00**	**1.00**
		SNV	1.00	1.00	0.98	0.97
		SG	0.99	0.98	1.00	1.00
	GM	**None**	**0.97**	**0.96**	**1.00**	**1.00**
		1D	0.94	0.91	1.00	1.00
		**2D**	**0.97**	**0.96**	**1.00**	**1.00**
		SNV	0.86	0.78	0.81	0.71
		SG	0.97	0.96	0.94	0.90
RSVM	BM	None	0.98	0.97	0.90	0.86
		**1D**	**1.00**	**1.00**	**0.99**	**0.99**

	2D	1.00	1.00	0.98	0.96
	SNV	0.99	0.98	0.79	0.68
	SG	0.99	0.98	0.91	0.86
CM	None	0.99	0.98	1.00	1.00
	1D	0.99	0.98	1.00	1.00
	**2D**	**1.00**	**1.00**	**1.00**	**1.00**
	SNV	0.99	0.99	0.93	0.89
	SG	0.99	0.98	1.00	1.00
GM	None	0.94	0.91	1.00	1.00
	**1D**	**0.96**	**0.93**	**1.00**	**1.00**
	2D	0.96	0.93	0.94	0.90
	SNV	0.67	0.48	0.44	0.05
	SG	0.94	0.91	1.00	1.00

[1]PLS-DA = partial least squares discriminant analysis; LSVM=svmLinear(support vector machine with kernel); RSVM=svmRadial(support vector machine with radial basis function kernel).

[2]BM= buffola milk; GM= goat milk; CM= camel milk.

[3]1D = first-order derivative , 2D = second-order derivative , SNV = Standard Normal Variate, and SG = Savitzky–Golsy convolution smoothing.

[4]Acc= Accuarcy.

Table S7 Comparison of the partial least squares (PLS) regression, and 11 modern statistical machine learning algorithm of the Fourier transform infrared spectra for determining the adulterated cow milk level in buffalo milk

**Modeling [1]**	**Preprocessing[2]**	**Calibration set[3]**				**Validation set[3]**			
		**RMSEc**	**MAEc**	**Rc²**	**RPDc**	**RMSEv**	**MAEv**	**Rv²**	**RPDv**
PLSR	None	7.16	5.60	0.80	2.24	8.00	6.27	0.73	1.95
	1D	6.63	5.21	0.83	2.42	9.22	6.92	0.67	1.69
	2D	6.66	5.20	0.83	2.41	8.15	6.37	0.73	1.91
	**SNV**	**6.53**	**4.89**	**0.83**	**2.46**	**7.56**	**5.83**	**0.76**	**2.06**
	SG	7.46	5.85	0.78	2.15	8.45	6.62	0.71	1.84
LSVM	None	7.70	4.98	0.81	2.08	8.63	6.15	0.73	1.80
	1D	6.80	4.76	0.84	2.36	9.26	6.46	0.65	1.68
	2D	6.48	4.66	0.84	2.48	9.02	6.50	0.68	1.73
	**SNV**	**6.68**	**4.66**	**0.84**	**2.40**	**7.88**	**5.87**	**0.74**	**1.98**
	SG	7.96	5.31	0.79	2.01	8.75	6.35	0.71	1.78
RSVM	None	6.11	3.31	0.86	2.63	8.79	5.69	0.70	1.77
	1D	5.13	2.82	0.90	3.13	7.62	5.15	0.76	2.04
	**2D**	**4.60**	**2.51**	**0.92**	**3.49**	**7.21**	**5.12**	**0.79**	**2.16**
	SNV	5.45	3.03	0.89	2.95	8.29	5.64	0.72	1.88
	SG	6.33	3.45	0.85	2.53	8.89	5.74	0.69	1.75
SSR	None	9.85	7.39	0.64	1.63	9.61	7.50	0.62	1.62

	1D	7.28	5.58	0.80	2.20	8.90	6.42	0.68	1.75
	2D	7.33	5.62	0.79	2.19	8.47	6.54	0.70	1.84
	**SNV**	**8.33**	**6.27**	**0.74**	**1.93**	**8.22**	**6.38**	**0.72**	**1.89**
	SG	10.26	7.68	0.61	1.56	9.97	7.77	0.59	1.56
PPR	**None**	**2.75**	**1.23**	**0.97**	**5.84**	**7.37**	**3.73**	**0.77**	**2.11**
	1D	2.66	1.09	0.97	6.04	7.49	3.71	0.77	2.08
	2D	2.73	1.16	0.97	5.87	8.39	4.65	0.71	1.86
	SNV	2.86	1.31	0.97	5.61	7.66	4.18	0.76	2.03
	SG	3.19	1.47	0.96	5.03	7.44	3.90	0.77	2.09
CART	None	11.65	8.90	0.47	1.38	12.15	8.90	0.40	1.28
	1D	7.33	4.61	0.79	2.19	12.83	7.63	0.42	1.21
	2D	7.83	4.74	0.76	2.05	13.25	8.25	0.34	1.18
	SNV	8.30	4.81	0.73	1.93	13.95	8.81	0.32	1.12
	**SG**	**8.21**	**4.90**	**0.74**	**1.95**	**11.51**	**6.80**	**0.49**	**1.35**
BRNN	None	4.79	3.15	0.91	3.35	6.15	4.11	0.84	2.53
	1D	4.88	3.26	0.91	3.29	6.11	4.20	0.85	2.55
	2D	5.17	3.44	0.90	3.10	6.12	4.17	0.85	2.54
	SNV	4.90	3.21	0.91	3.27	6.09	4.10	0.85	2.56
	**SG**	**4.94**	**3.27**	**0.91**	**3.25**	**6.02**	**4.09**	**0.85**	**2.59**
RR	None	9.88	7.38	0.64	1.62	9.62	7.50	0.62	1.62
	1D	7.42	5.65	0.79	2.16	8.58	6.50	0.70	1.81
	2D	7.29	5.59	0.80	2.20	8.39	6.53	0.71	1.86
	**SNV**	**8.24**	**6.23**	**0.75**	**1.95**	**8.28**	**6.46**	**0.71**	**1.88**
	SG	10.37	7.74	0.60	1.55	10.00	7.76	0.59	1.56
EN	None	6.96	5.42	0.81	2.31	7.83	6.04	0.74	1.99
	1D	6.40	5.02	0.84	2.51	8.79	6.48	0.69	1.77
	2D	6.28	4.89	0.85	2.56	8.05	6.13	0.74	1.93
	**SNV**	**6.41**	**4.90**	**0.84**	**2.50**	**7.45**	**5.69**	**0.77**	**2.09**
	SG	7.18	5.63	0.80	2.24	8.15	6.27	0.72	1.91
LASSO	None	6.41	5.07	0.84	2.50	7.72	6.04	0.75	2.02
	1D	6.41	5.03	0.84	2.50	8.75	6.47	0.69	1.78
	2D	6.21	4.83	0.85	2.58	8.08	6.16	0.73	1.93
	**SNV**	**6.32**	**4.83**	**0.84**	**2.54**	**7.36**	**5.63**	**0.77**	**2.12**
	SG	7.29	5.69	0.79	2.20	8.12	6.28	0.73	1.92
RF	None	4.47	2.78	0.93	3.59	9.38	6.24	0.63	1.66
	1D	4.15	2.63	0.95	3.87	8.83	6.11	0.68	1.76
	**2D**	**3.77**	**2.35**	**0.96**	**4.25**	**8.25**	**5.70**	**0.72**	**1.89**
	SNV	4.79	3.12	0.93	3.35	10.62	7.22	0.53	1.47
	SG	4.51	2.77	0.93	3.56	9.26	6.14	0.64	1.68
GBM	None	4.88	3.08	0.91	3.29	9.24	6.20	0.65	1.68
	**1D**	**4.15**	**2.82**	**0.93**	**3.87**	**8.81**	**6.01**	**0.70**	**1.77**
	2D	4.22	2.69	0.93	3.80	10.57	7.27	0.55	1.47
	SNV	4.22	2.69	0.93	3.80	10.57	7.27	0.55	1.47
	SG	5.59	3.72	0.88	2.87	9.62	6.69	0.62	1.62

PCA+	None	6.00	4.01	0.86	2.67	10.17	6.55	0.63	1.53
BRNN	**1D**	**5.74**	**3.88**	**0.87**	**2.80**	**5.42**	**3.65**	**0.88**	**2.87**
	2D	5.75	3.77	0.87	2.79	6.47	4.38	0.83	2.41
	SNV	6.48	4.14	0.84	2.48	7.20	4.52	0.79	2.16
	SG	7.29	5.07	0.79	2.20	8.37	5.84	0.74	1.86

[1]PLSR=Partial least squares regression, LSVM=svmLinear(support vector machine with kernel), RSVM=svmRadial(support vector machine with radial basis function kernel), SSR= Spike and Slab Regression, PPR= Projection Pursuit Regression, CART= Classification and Regression Tree, BRNN=Bayesian Regularized Neural Networks, RR= Ridge Regression, EN= Elastic net Regression, LASSO= Least Absolute Shrinkage and Selection Operator; RF= Random Forest, and GBM= Gradient Boosting Machine.

[2]1D = first-order derivative , 2D = second-order derivative , SNV = Standard Normal Variate, and SG = Savitzky–Golsy convolution smoothing.

[3]RMSE= the Root Mean Square Error, MAE= the Mean Absolute Error, R^2= the coefficient of determination, and RPD= the ratio performance deviation.

Table S8 Comparison of the partial least squares (PLS) regression, and 11 modern statistical machine learning algorithm of the Fourier transform infrared spectra for determining the adulterated water level in buffalo milk

**Modeling[1]**	**Preprocessing[2]**	**Calibration set[3]**				**Validation set[3]**			
		**RMSEC**	**MAEc**	**R_c^2**	**RPDc**	**RMSEV**	**MAEv**	**R_v^2**	**RPDv**
PLSR	**None**	**2.06**	**1.31**	**0.99**	**9.19**	**2.25**	**1.50**	**0.99**	**8.39**
	1D	2.17	1.41	0.99	8.72	2.32	1.64	0.99	8.15
	2D	2.20	1.45	0.99	8.61	2.50	1.73	0.98	7.56
	SNV	2.66	1.94	0.98	7.13	3.02	2.22	0.97	6.25
	SG	2.13	1.37	0.99	8.90	2.33	1.57	0.99	8.12
LSVM	None	2.11	1.32	0.99	8.96	2.23	1.48	0.99	8.48
	**1D**	**2.09**	**1.32**	**0.99**	**9.08**	**2.20**	**1.48**	**0.99**	**8.58**
	2D	2.14	1.36	0.99	8.85	2.41	1.64	0.98	7.83
	SNV	2.26	1.57	0.99	8.36	2.52	1.76	0.98	7.50
	SG	2.19	1.37	0.99	8.64	2.25	1.49	0.99	8.41
RSVM	None	2.16	1.53	0.99	8.77	2.94	2.01	0.98	6.43
	**1D**	**1.66**	**1.29**	**0.99**	**11.40**	**2.76**	**1.86**	**0.98**	**6.85**
	2D	1.55	1.25	0.99	12.22	3.00	2.06	0.98	6.31
	SNV	1.94	1.59	0.99	9.74	6.51	4.41	0.89	2.90
	SG	2.16	1.52	0.99	8.75	2.95	2.00	0.98	6.40
SSR	None	3.00	1.99	0.97	6.31	3.01	2.15	0.98	6.27
	**1D**	**2.39**	**1.50**	**0.98**	**7.91**	**2.47**	**1.73**	**0.98**	**7.64**
	2D	2.35	1.49	0.98	8.06	2.57	1.73	0.98	7.34
	SNV	3.54	2.56	0.96	5.34	3.91	2.90	0.96	4.84
	SG	3.04	2.05	0.97	6.23	2.92	2.08	0.98	6.46
PPR	None	0.78	0.34	1.00	24.19	2.35	0.95	0.99	8.03
	1D	0.74	0.32	1.00	25.58	2.44	0.96	0.98	7.74

	2D	0.74	0.32	1.00	25.49	2.39	0.97	0.98	7.92
	SNV	0.66	0.29	1.00	28.56	2.40	0.83	0.98	7.87
	**SG**	**1.03**	**0.30**	**1.00**	**18.30**	**1.67**	**0.59**	**0.99**	**11.30**
CART	None	2.36	0.78	0.98	8.03	4.20	1.39	0.95	4.49
	1D	1.85	0.57	0.99	10.25	3.69	1.28	0.96	5.12
	**2D**	**1.91**	**0.57**	**0.99**	**9.90**	**3.23**	**1.09**	**0.97**	**5.86**
	SNV	9.10	6.19	0.77	2.08	14.69	10.78	0.42	1.29
	SG	2.33	0.76	0.98	8.11	4.18	1.39	0.95	4.52
BRNN	None	1.75	1.10	0.99	10.85	2.15	1.37	0.99	8.78
	1D	1.73	1.10	0.99	10.92	2.14	1.38	0.99	8.82
	2D	1.10	0.78	1.00	17.27	3.01	1.55	0.98	6.28
	SNV	1.03	0.81	1.00	18.44	2.30	1.52	0.99	8.20
	**SG**	**1.80**	**1.15**	**0.99**	**10.52**	**2.12**	**1.32**	**0.99**	**8.92**
RR	None	3.29	2.30	0.97	5.75	3.17	2.33	0.97	5.96
	**1D**	**2.61**	**1.71**	**0.98**	**7.25**	**2.58**	**1.86**	**0.98**	**7.33**
	2D	2.55	1.63	0.98	7.43	2.71	1.84	0.98	6.96
	SNV	4.45	3.21	0.95	4.25	4.93	3.81	0.94	3.84
	SG	3.39	2.40	0.97	5.59	3.18	2.37	0.97	5.93
EN	None	2.52	1.66	0.98	7.53	2.62	1.79	0.98	7.22
	**1D**	**2.24**	**1.43**	**0.99**	**8.46**	**2.37**	**1.66**	**0.98**	**7.97**
	2D	2.26	1.44	0.99	8.36	2.50	1.69	0.98	7.55
	SNV	2.47	1.80	0.98	7.68	2.91	2.07	0.98	6.50
	SG	2.47	1.61	0.98	7.65	2.57	1.76	0.98	7.35
LASSO	None	3.32	2.29	0.97	5.71	3.30	2.36	0.97	5.73
	**1D**	**2.43**	**1.54**	**0.98**	**7.79**	**2.50**	**1.76**	**0.98**	**7.57**
	2D	2.30	1.47	0.99	8.23	2.55	1.72	0.98	7.40
	SNV	2.48	1.81	0.98	7.64	2.92	2.09	0.98	6.46
	SG	2.90	1.95	0.98	6.53	2.80	2.01	0.98	6.76
RF	None	1.15	0.51	1.00	16.45	2.70	1.28	0.98	6.99
	1D	1.05	0.45	1.00	18.09	2.76	1.36	0.98	6.84
	**2D**	**1.10**	**0.52**	**1.00**	**17.26**	**2.64**	**1.41**	**0.98**	**7.17**
	SNV	3.97	2.90	0.97	4.78	10.83	7.90	0.71	1.74
	SG	1.25	0.54	1.00	15.09	2.73	1.24	0.98	6.92
GBM	None	0.17	0.09	1.00	114.33	3.15	1.58	0.97	6.01
	**1D**	**0.18**	**0.07**	**1.00**	**104.93**	**2.51**	**1.35**	**0.98**	**7.53**
	2D	0.35	0.15	1.00	53.94	2.57	1.42	0.98	7.35
	**SNV**	0.69	0.54	1.00	27.31	7.57	5.21	0.85	2.50
	SG	0.16	0.08	1.00	116.99	3.27	1.50	0.97	5.78
PCA+PPR	None	2.55	1.33	0.98	7.42	2.73	1.57	0.98	6.91
	**1D**	1.23	0.44	1.00	15.40	1.70	0.68	0.99	11.10
	2D	1.38	0.57	0.99	13.68	2.08	0.91	0.99	9.09
	**SNV**	2.66	1.95	0.98	7.12	2.94	2.17	0.98	6.43
	SG	2.47	1.25	0.98	7.65	2.78	1.61	0.98	6.79

[1]PLSR=Partial least squares regression, LSVM=svmLinear(support vector machine

with kernel), RSVM=svmRadial(support vector machine with radial basis function kernel), SSR= Spike and Slab Regression, PPR= Projection Pursuit Regression, CART= Classification and Regression Tree, BRNN=Bayesian Regularized Neural Networks, RR= Ridge Regression, EN= Elastic net Regression, LASSO= Least Absolute Shrinkage and Selection Operator; RF= Random Forest, and GBM= Gradient Boosting Machine.

[2]1D = first-order derivative , 2D = second-order derivative , SNV = Standard Normal Variate, and SG = Savitzky–Golsy convolution smoothing.

[3]RMSE= the Root Mean Square Error, MAE= the Mean Absolute Error, R^2= the coefficient of determination, and RPD= the ratio performance deviation.
